# Clinical application of machine learning models in patients with prostate cancer before prostatectomy

**DOI:** 10.1186/s40644-024-00666-y

**Published:** 2024-02-08

**Authors:** Adalgisa Guerra, Matthew R. Orton, Helen Wang, Marianna Konidari, Kris Maes, Nickolas K. Papanikolaou, Dow Mu Koh

**Affiliations:** 1 Department of Radiology, Hospital da Luz Lisbon, Rua Fernando Curado Ribeiro, 2, 7º esq, 1495-094 Algés, Lisboa, Portugal; 2 Royal Marsden Hospital NHS Foundation Trust, London, England; 3 Royal Surrey County Hospital NSH Foundation Trust, Royal Marsden Hospital NHS Foundation Trust, London, England; 4 Department of Urology, Hospital da Luz Lisbon, Lisbon, Portugal

**Keywords:** Prostate cancer, Extracapsular extension, MRI, Radiomics, Machine learning

## Abstract

**Background:**

To build machine learning predictive models for surgical risk assessment of extracapsular extension (ECE) in patients with prostate cancer (PCa) before radical prostatectomy; and to compare the use of decision curve analysis (DCA) and receiver operating characteristic (ROC) metrics for selecting input feature combinations in models.

**Methods:**

This retrospective observational study included two independent data sets: 139 participants from a single institution (training), and 55 from 15 other institutions (external validation), both treated with Robotic Assisted Radical Prostatectomy (RARP). Five ML models, based on different combinations of clinical, semantic (interpreted by a radiologist) and radiomics features computed from T2W-MRI images, were built to predict extracapsular extension in the prostatectomy specimen (pECE+). DCA plots were used to rank the models’ net benefit when assigning patients to prostatectomy with non-nerve-sparing surgery (NNSS) or nerve-sparing surgery (NSS), depending on the predicted ECE status. DCA model rankings were compared with those drived from ROC area under the curve (AUC).

**Results:**

In the training data, the model using clinical, semantic, and radiomics features gave the highest net benefit values across relevant threshold probabilities, and similar decision curve was observed in the external validation data. The model ranking using the AUC was different in the discovery group and favoured the model using clinical + semantic features only.

**Conclusions:**

The combined model based on clinical, semantic and radiomic features may be used to predict pECE + in patients with PCa and results in a positive net benefit when used to choose between prostatectomy with NNS or NNSS.

**Supplementary Information:**

The online version contains supplementary material available at 10.1186/s40644-024-00666-y.

## Background

Prostate cancer (PCa) is the second most commonly diagnosed malignancy in men and is the second leading cause of mortality from cancer [1]. Radical prostatectomy is a well-established treatment for managing localized PCa, and the goal is to achieve a negative surgical margin while preserving urinary continence and erectile function. As such, accurate preoperative staging is of great importance for guiding treatment [2].

Multiparametric magnetic resonance imaging (mpMRI), is the recommended imaging method for tumour detection and for differentiating advanced cancers with extracapsular extension (ECE) from localized disease [3, 4]. The use of mpMRI combined with traditional clinicopathological-based risk nomograms is recommended before prostatectomy to determinate the need for nerve-sparing surgery (NSS) and pelvic lymphadenectomy [2, 5, 6].

The MRI-based assessment of ECE reported in the literature by Mehralivand et al. [7], the European Society of Urogenital Radiology (ESUR) score [8], a subjectively measured Likert scale [9], and measurement of TCCL (tumour capsular contact length) were recently compared by Park et al. [10], in a group of 301 patients (43% with pathologic ECE). The study showed sensitivity between 68 and 82% for extraprostatic extension detection [10]. These MRI scoring schemes demonstrated fair diagnostic performance, substantial agreement and association with histopathologic tumour extension [11], however, considerable observer variabilities remain a significant challenge in utilising these mpMRI-based scores [10]^,^ [11].

Machine learning (ML) applications in patients with prostate cancer remain an active research area focusing primarily on automatic segmentation, detection and localization, and assessment of disease aggressiveness using mpMRI [12]^,^ [13]. At present, only a few studies have introduced ML models to predict pECE+ (presence of ECE in pathology specimens) in PCa staging by mpMRI. Most used combinations of radiomic features were extracted from MRI T2 weighted images with semantic and clinical features to predict ECE [14–20]. To the best of our knowledge, clinically accepted and validated algorithms to predict pECE + obtained from MRI features for use in preoperative PCa surgical decision-making have not been developed to date. The metric usually used to guide model selection is area under the curve (AUC), which does not account for the specific use case. Decision curve analysis (DCA) [21] is a method of assessing the clinical utility of ML predictive models because it enables assessment of the net benefit achieved when a predictive model is used in a specified scenario.

**Fig. 1:**
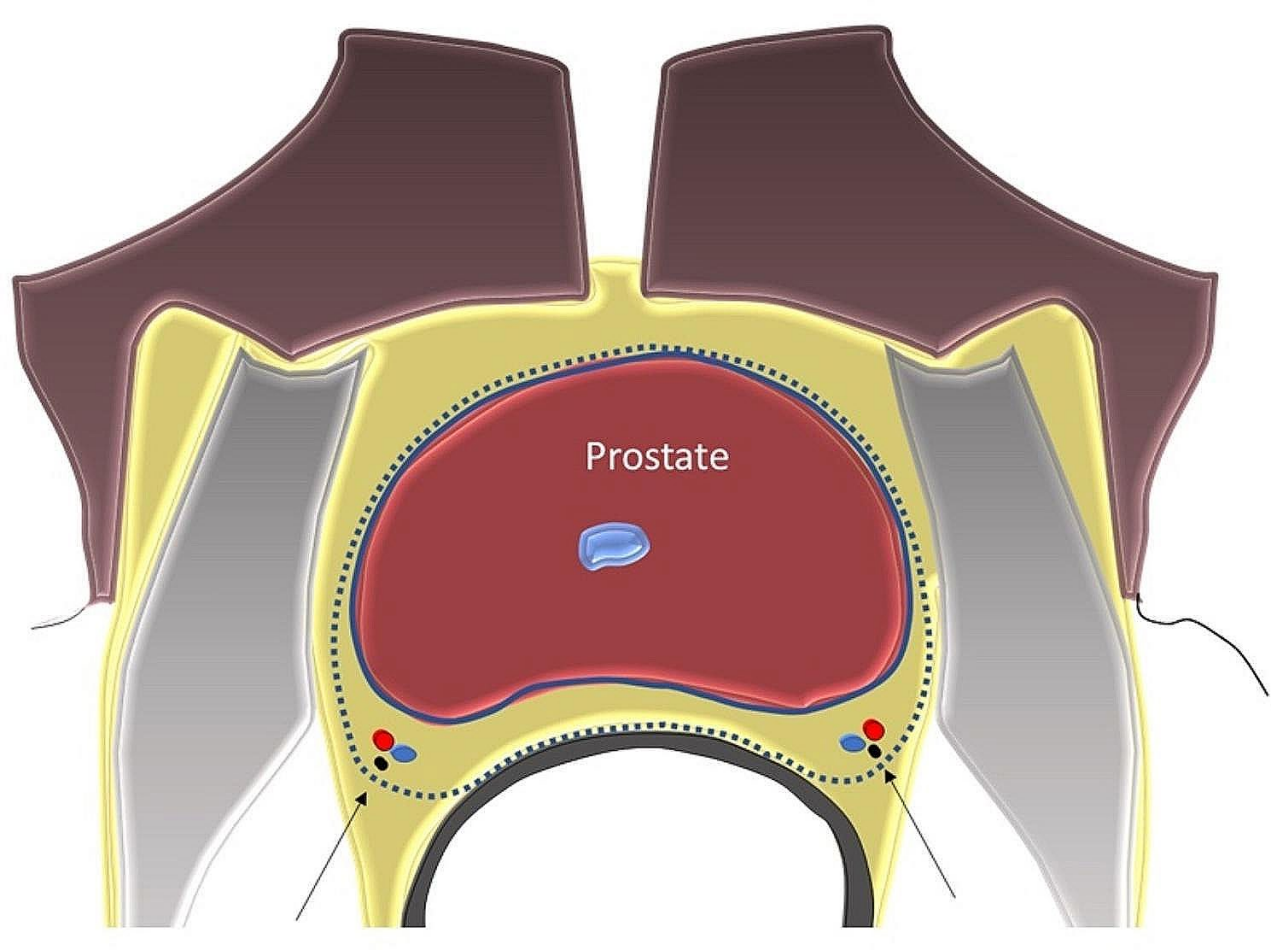
Schema of prostatectomy types Peri-prostatic nerves (arrow) near the prostate capsule are not dissected (blue line) and only the prostate and capsule are removed in NSS (nerve-sparing surgery) prostatectomy. In the NNSS (non-nerve-sparing surgery) the peri-prostatic nerves and some extracapsular area are removed (dashed line)

The objective of this study was to develop ML predictive models for use in PCa surgical decision-making for a specific clinical use-case: to choose between the use of nerve-sparing surgery (NSS) and non-nerve sparing surgery (NNSS) when performing prostatectomy in patients with PCa (Fig. 1).

## Methods

### Participants characteristics

**Fig. 2:**
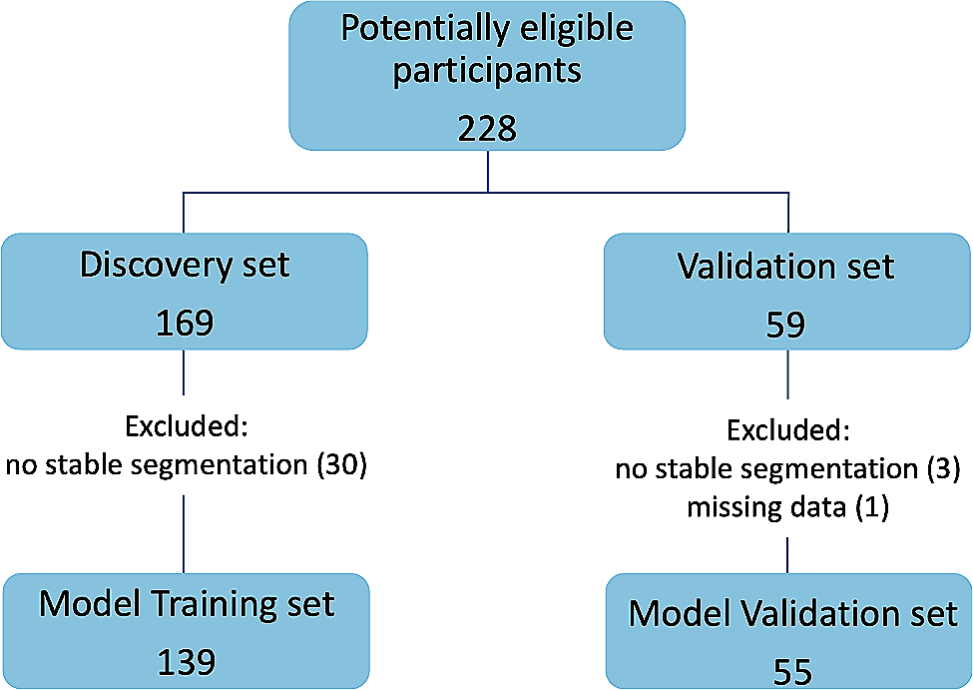
Flowchart of study cohort selection

Two independent data sets were available: a) discovery–single institution, Hospital da Luz (HdL), *N* = 139, used for training (underwent the institutional MRI protocol-Supplements Table S1; b) validation–multi-centre (15 external institutions), *N* = 55, used for testing. This cohort was part of a previously published predictive model [22] without radiomics analysis (Fig. 2).

All participants included in this study (discovery and validation groups) underwent Robotic Assisted Radical Prostatectomy (RARP) with pathologically confirmed PCa on prostate biopsy and index lesion PIRADS > 2 (PI-RADS v2) on MRI. A uropathologist (JC) with 10 years’ experience analysed all surgically resected prostate gland specimens using the same protocol, including the determination of ECE status. Matched cases, correlated pathology and radiology results from the pathologist (JC) and radiologist (AG) were included.

### Data upload and curation

MRI DICOM images were pseudonymized and transferred to a research PACS based on the extensible Neuroimaging Archive Toolkit (XNAT) platform [23], which served as the principal repository for image curation and analysis.

### Segmentation

One radiologist (AG, ten years’ experience) manually segmented the region of interest (index lesion) for all cases in both data sets. A second radiologist (MK, three years’ experience) independently segmented a randomized selection of 30 cases from the discovery dataset (stratified on lesion size) to enable radiomic feature reproducibility to be determined.

### Radiomics extraction

T2W MR images were interpolated to standard voxel size (0.5 × 0.5 × 3 mm) and z-score image normalization was applied. The normalized image intensities were quantized to 64 bins using the built-in uniform quantization method in Pyradiomics [24] and 107 features were calculated.

### Semantic and clinical features

**Fig. 3:**
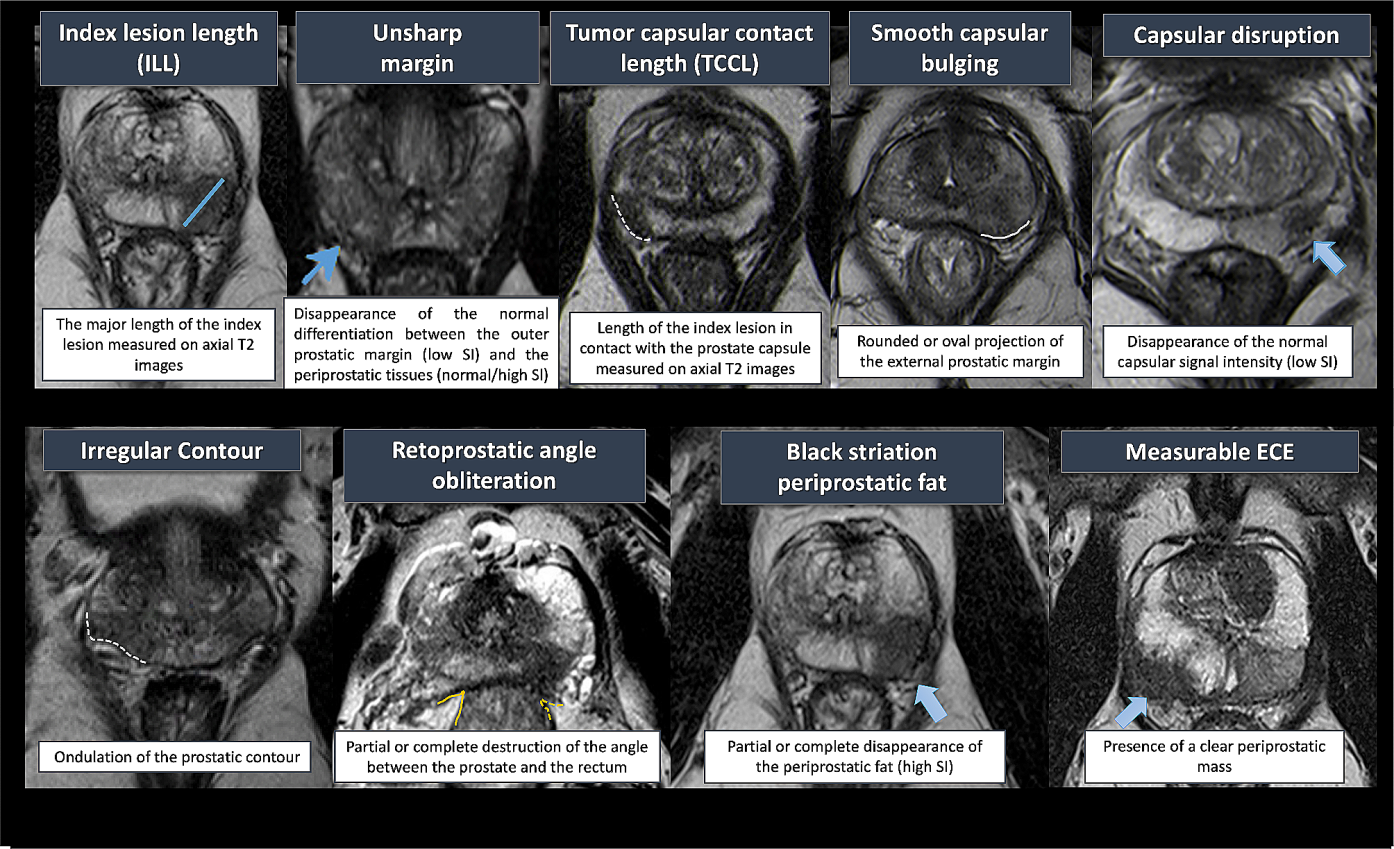
MRI Semantic features for detection of ECE+ This figure illustrates the eight semantic features, interpreted by radiologists, used in semantic model to predict pECE+, on axial T2WI. The measurable ECE was not used in semantic model and it is considered alone in another model as explained in the text

The first radiologist (AG) classified the index lesion for both data sets using eight semantic features (Fig. 3). The second radiologist (MK) independently classified the semantic features for the whole discovery set to enable feature reproducibility to be determined. Both radiologists also classified each lesion as having measurable ECE (mECE), i.e., the presence of a clear periprostatic extension.

Three clinical features were obtained from the electronic patient record (EPR): Gleason score, prostate volume and PSA. AG classified the index lesion in accordance with PIRADS-v2 [25]. Due to the small sample size, Gleason score was grouped into two classes based on tumour aggressiveness: low = with Gleason score of 6(3 + 3) or 7(3 + 4); high = including cases with a Gleason score of 7(4 + 3) or above. The PIRADS score was treated as a categorical variable to enable the predictive model to fit a non-proportional effect to this feature.

### Feature reproducibility

Inter-observer variability was assessed using the intraclass correlation coefficient (ICC) [26], for radiomics and continuous semantic features (lesion size and tumour capsular contact length (TCCL), and Cohen’s kappa for the binary semantic features. Radiomic features with ICC > 0.75 were used for model building and the remaining features were discarded. All semantic features were used for model building, and their reproducibility estimates were used to identify features which are most likely to adversely impact the stability of the ECE status predictions, and therefore which features would benefit most from further standardization efforts.

### Model discovery and validation

Models were built from the discovery data using the following four combinations of the three feature sets: i)clinical; ii) CS:clinical + semantic; iii) CR:clinical + radiomics; iv) CSR:clinical + semantic + radiomics. All combinations include the clinical features because they are routinely obtained for all participants as part of their standard of care.

For the two models that include radiomics features, a hierarchical feature reduction scheme [27] was used to remove correlated features with Spearman’s correlation > 0.9.

Models were built using logistic regression (LR), and LASSO regularization was used for feature selection in the three models that included semantic or radiomic features. The LASSO regularization parameter was tuned using 10-fold cross-validation (CV) over a log-spaced grid (20 values, 10^− 4^–10^4^), and each input feature was z-score normalized. A fifth model (univariate LR) was built using the mECE feature, which enabled baseline ROC and DCA curves to be constructed.

**Fig. 4:**
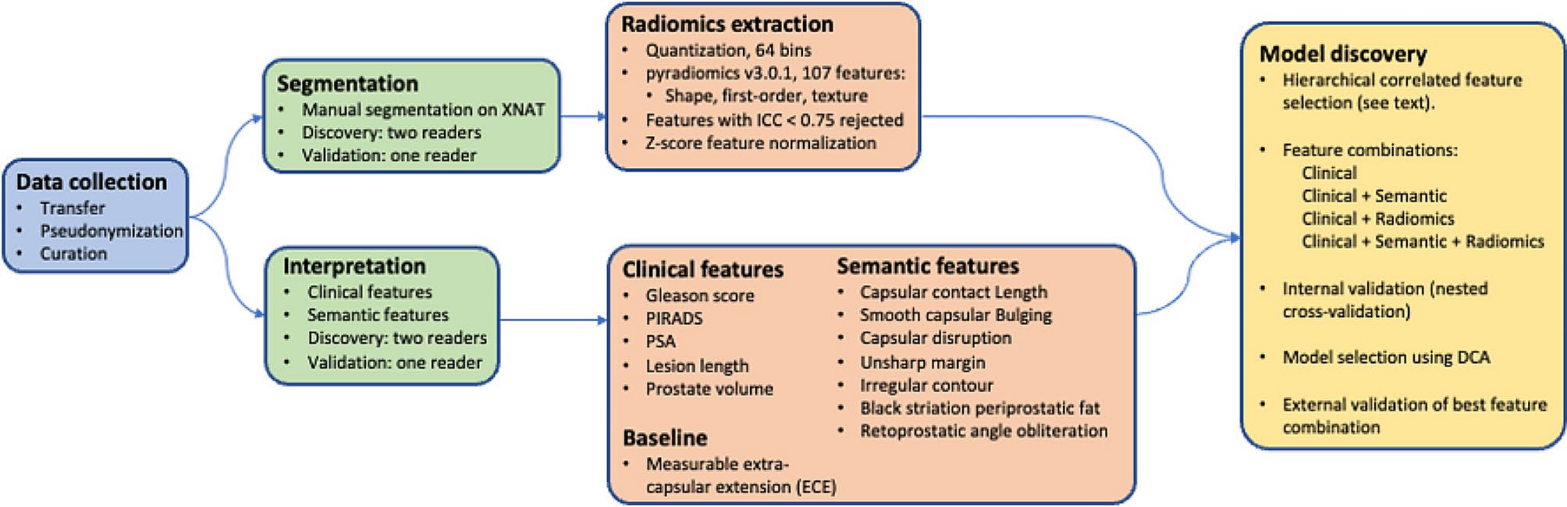
Model development pipeline

Performance metrics for the discovery data set were estimated using a 10-fold CV repeated 100x, such that the parameter tuning CV was nested inside the performance estimation CV. Performance indicators included accuracy, F1-score, AUC, the ROC curve, and the DCA net-benefit curve [21], and these were computed for each of the outer CV splits and averaged to generate the final values and plots. The DCA net-benefit curves were used to select the final model that was tested in the validation data. An interpretation of this model was obtained using SHAP [28] analysis (SHapley Additive exPlanations), which explains the model predictions by computing the contribution of each feature to the overall risk prediction for each patient. The DCA and ROC curves were calculated for the validation data using the final model. The model development pipeline is shown in Fig. 4.

## Results

### Participants characteristics

Table 1 summarizes the clinical and semantic feature distributions of both data sets. There were no statistically significant differences between the discovery and validation data sets (*p* > 0.05), except smooth capsular bulging (*p* = 0.03). However, this feature was not selected in any of the models evaluated in the validation data. A majority of participants did not have ECE detected in their surgical specimens (74.1% and 65.5% in the discovery and validation groups), and we conclude that the populations and MRI examinations in the two data sets are comparable.

**Table 1:** Data distributions of the clinical and semantic features in the discovery and validation data sets. Binary semantic features have the counts for absent/present, (values in parentheses are percentages), and mean +/ sd is given for continuous features. *P*-values comparing the discovery and test distributions are computed using Fisher’s exact test for binary features (and PIRADS), and unpaired t-tests are used for continuous features

	Feature	Discovery (*N* = 139)		Test (*N* = 55)		*p*-value
Clinical	Gleason (low/high)	97/42	(69.8/30.2)	33/22	(60.0/40.0)	0.24
	PIRADS (3/4/5)	6/75/58	(4.3/54.0/41.7)	3/33/19	(5.5/60.0/34.5)	0.68
	PSA	7.12 +/- 4.02		6.97 +/- 5.20		0.45
	Major Length Index	14.3 +/- 5.7		14.1 +/- 6.8		0.90
	Prostate Volume	43.0 +/- 21.3		48.3 +/- 23.8		0.084
Semantic	Capsular Contact Length	12.5 +/- 8.8		12.8 +/- 11.2		0.85
	Smooth Capsular Bulging	55/84	(39.6/60.4)	32/23	(58.2/41.8)	0.03
	Capsular Disruption	71/68	(51.1/48.9)	34/21	(61.8/38.2)	0.20
	Unsharp Margin	64/75	(46.0/54.0)	32/23	(58.2/41.8)	0.15
	Irregular Contour	80/59	(57.6/42.4)	34/21	(61.8/38.2)	0.63
	Black striation Periprostatic Fat	110/29	(79.1/20.9)	44/11	(80.0/20.0)	1.00
	Retoprostatic Angle Obliteration	130/9	(93.5/6.5)	49/6	(89.1/10.9)	0.37
Baseline	Measurable ECE	119/20	(85.6/14.4)	48/7	(87.3/12.7)	1.00
Target	Pathological ECE	103/36	(74.1/25.9)	36/19	(65.5/34.5)	0.29

### Model performance comparisons

**Fig. 5:**
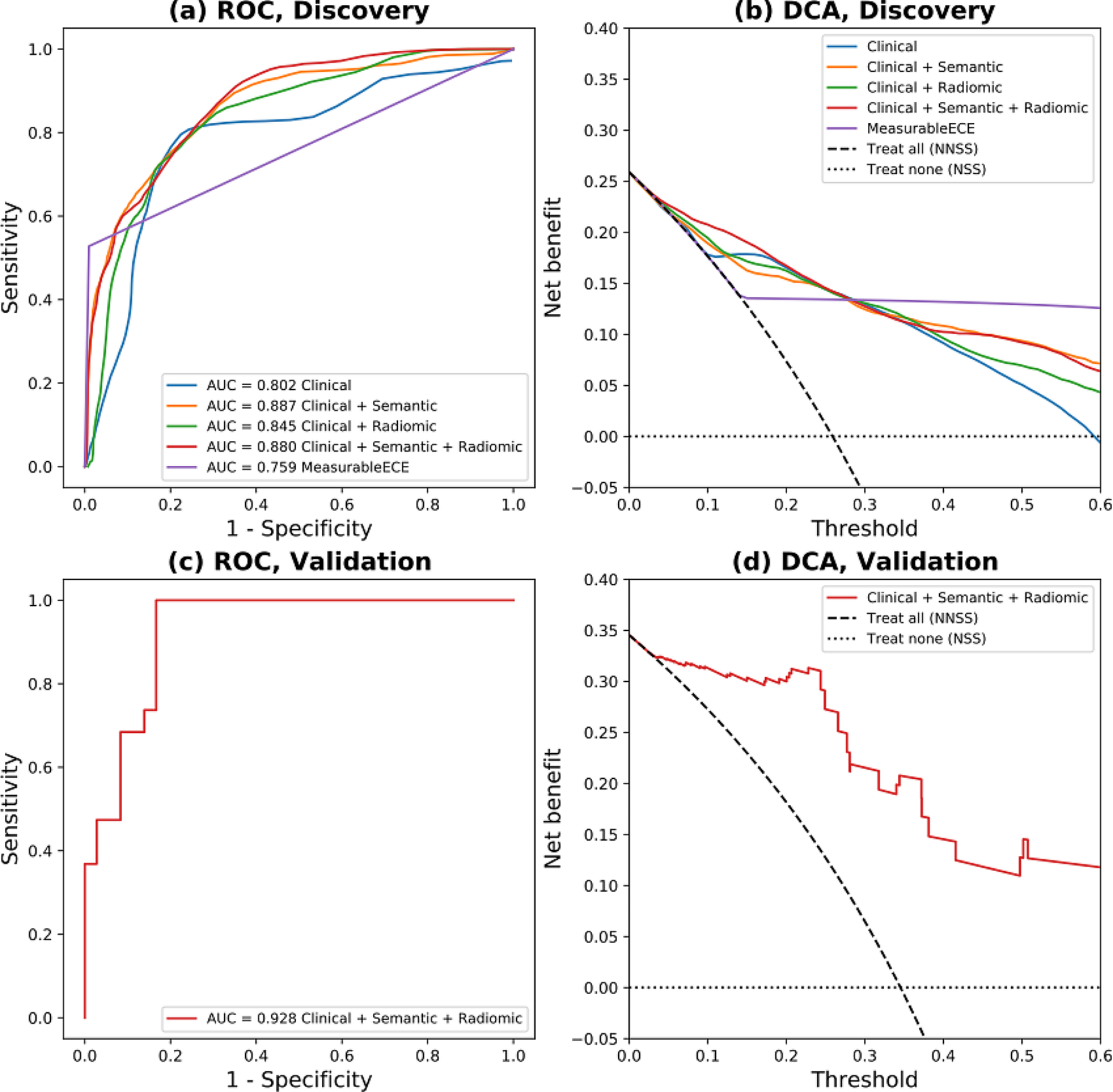
ROC and DCA plots for the four multivariate predictive models for ECE+(blue, orange, green, red lines) and the univariate model derived from mECE (purple line) in participants with PCa. Panels (**a**), ROC and (**b**), DCA are for the discovery data set and panels (**c**) and (**d**) are for the validation data set, respectively. The DCA plots also include lines for the net benefit when all participants receive non-nerve-sparing surgery (NNSS) and when no participants receive NNSS (i.e. when all participants receive nerve-sparing surgery-NSS). The net benefit is equal to or higher than both lines for all models. The x-axis of the DCA plots is the threshold of the risk predicted by the model at which NNSS would be indicated. A vital aspect of the DCA concept is that this threshold is directly related to the ratio of the cost associated with false negative and false positive predictions– low values of the threshold correspond to the use case where failing to give NNSS (with curative intent) is more costly than the complications that may arise from using NNSS

Model performance metrics (AUC, accuracy and F1 score) are given in Table 2 and the ROC and DCA curves are shown in Fig. 5. for the discovery and validation data. As previously mentioned, model selection was determined based on the DCA curves in the discovery data (Fig. 5b). Up to a threshold of 0.3, the net benefit of the CSR model (red line) is higher than the three other multivariate models and the univariate model derived from mECE. This selection of the CSR model as the final model is different from what would be obtained by using the performance metrics in Table 2, where the clinical + semantic model has higher values for all performance metrics compared to the other three multivariate models. The baseline univariate model derived from mECE had higher accuracy and F1 score, but this was at the expense of a lower AUC.

Table 2 shows that the accuracy and F1 scores of the CSR model are somewhat lower in the validation data. In contrast, the AUC is in fact higher for the validation data. Whilst this elevation is unusual, it is reasonable since the performance metrics are derived from two patient samples and are therefore influenced by random fluctuations related to patient variability. Although the validation AUC for the CSR model (0.928) is higher than the average AUC in the discovery data (0.880), it is smaller than 37% of the values from the 1000 cross-validation splits used to obtain the discovery mean AUC estimate.

**Table 2:** Performance metrics for the five predictive models in the discovery and validation data sets. Error limits are +/- 1 standard deviation across 1000 CV splits

	Metric	Measurable ECE	Clinical	Clinical + Semantic	Clinical + Radiomic	Clinical + Semantic + Radiomic
Discovery	AUC	0.759	0.802 ± 0.149	0.887 ± 0.102	0.845 ± 0.121	0.880 ± 0.101
	Accuracy	0.871	0.790 ± 0.102	0.835 ± 0.092	0.811 ± 0.099	0.833 ± 0.092
	F1	0.679	0.560 ± 0.232	0.625 ± 0.225	0.602 ± 0.222	0.623 ± 0.228
Validation	AUC					0.928
	Accuracy					0.782
	F1					0.6
	*p*-value					2.3 × 10^− 7^

### Model explanation via SHAP analysis

**Fig. 6:**
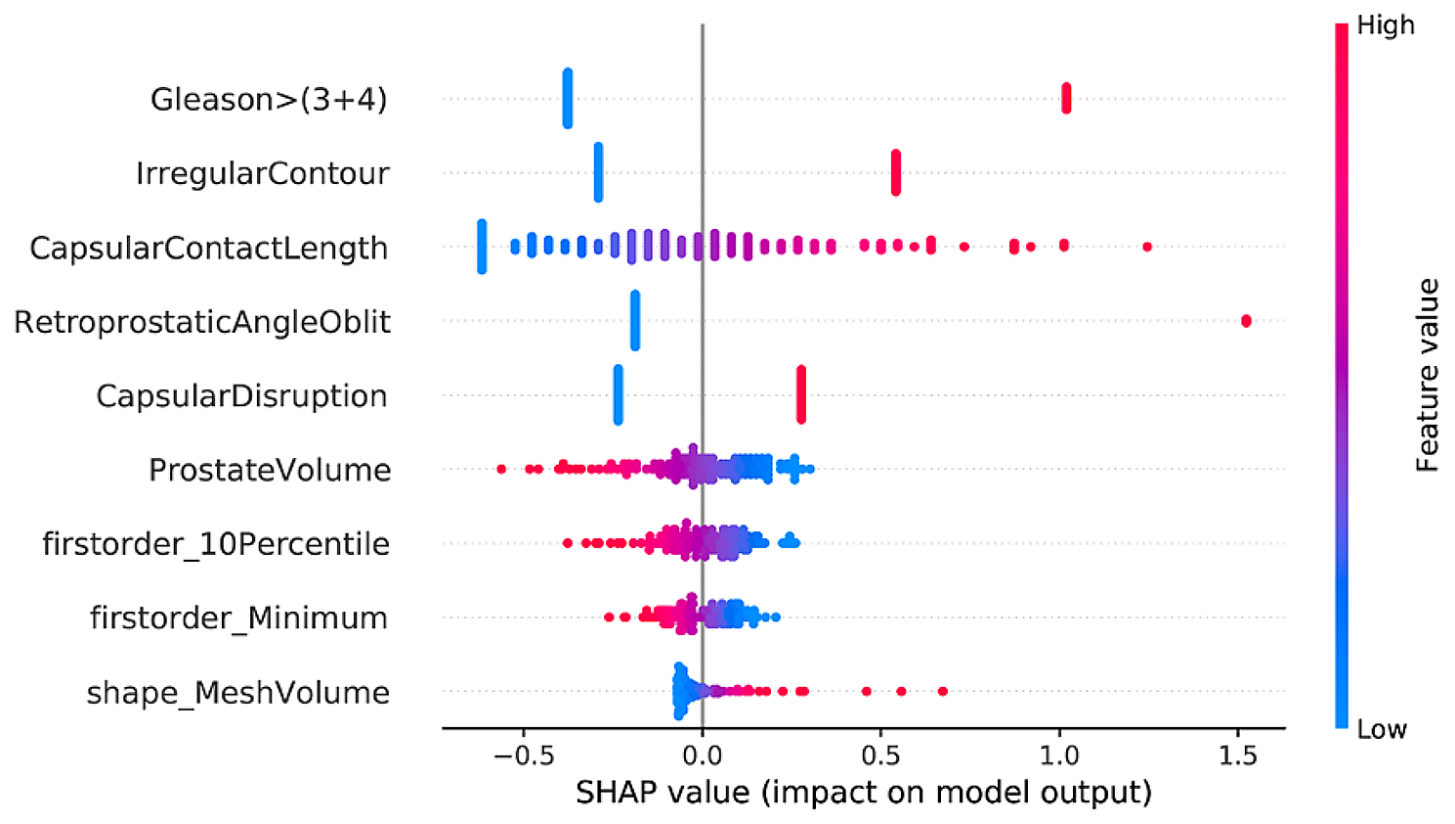
Beeswarm plot of SHAP values for the final model developed using clinical + semantic + radiomic features, which represents the influence of each feature when predicting pECE+. Blue dots imply low values for each feature, while red dots indicate high values, and positive SHAP values suggest a risk increase of pECE+, and vice versa for negative SHAP values

Figure 6 shows the SHAP beeswarm plot for the CSR model, where the most influential features (based on the average SHAP value across all participants) are at the top of the plot. For the top five features in this plot, high positive SHAP values are associated with high feature values, which indictes an increased risk of pECE + for participants with high Gleason scores, longer TCCL and positive findings for Irregular contour, retoprostatic angle obliteration and capsular disruption. TCCL was the reproducible semantic feature (supplements Table S2). Prostate volume appeared to have a protective effect (larger values are associated with lower ECE risk), and the clinical features PSA and PI-RADS score were not present in the model. Three radiomics features appeared in the model– the two first-order features (10Percentile and Minimum) indicated increased pECE + risk for lower values. In contrast, the shape feature (MeshVolume, i.e. the lesion volume) suggested a more significant pECE + risk for larger lesion volumes, and all three radiomics features were highly reproducible (supplements Table S3). None of the second-order (texture) radiomics features were present in the model.

## Discussion

We built five machine learning predictive models to detect pECE + and compared them for selecting NNSS if ECE + is predicted, as this has a better chance of controlling the disease than NSS. The five models were built using clinical tools and semantic features previously described by the first author [22], with the addition of new radiomics features derived from MRI images: i) clinical; ii) CS:-clinical + semantic; iii) CR-clinical + radiomics; iv) CSR-clinical + semantic + radiomics (according to an adequate pipeline criterion and with inter-reading agreement) and lastly v) univariate measurable ECE model. The CS model achieved the best AUC results in the discovery set and the CSR model was almost as good as CS in the discovery set (Table 2). This CSR model maintains good performance in the validation data, and has the advantage that radiomics features were included which were reproducible with ICC agreement > 75% between readers. From all festures in the CS, the TCCL achieve the best ICC (0,683) reproducible between readers. Our results align with previous ones supported in the literature, which proved that combining radiomics, clinical and semantic models to predict pECE + is more accurate than individual models [14, 15, 29–31]. This paper follows the previously published work by the lead author [22], where clinical + semantic features were used to develop a predictive model based on a classic logistic regression algorithm to predict pECE + with a good performance (AUC 90%). Based on ML methdology, the main clinical and semantic predictive features obtained were GS > (3 + 4) and TCCL, similar to the previously published results [22, 29, 30]. Furthermore, with the addition of a radiomics signature, we improved the reproducibility, reducing the subjective nature of the previous model, which relied on MRI conventional visual interpretation by radiologists.

At present, predictive signatures to detect pECE + have been published but these have not been considered against surgical decision making [29]. ^,^ [32] In this study, we have gone further to examine how our model could perform in real life and quantify the potential impact of using it to choose between NSS versus NNSS. Most surgeons advocate NSS for patients with pECE- to achieve lower morbidity from nerve damage, such as incontinence and erectile dysfunction, keeping high negative surgical margins (NSM). While patients with pECE + would benefit from NNSS to achieve NSM, despite the increase risk of morbidity from nerve damage and other surgical side-effects. The DCA method was used to compare the net benefit of all five predictive models to detect pECE+, also comparing to the “treat all” case (i.e., treating all patients with NNSS) and “treat none”(i.e., treating no patients with NNSS, meaning treat all patients with NSS as the default treatment), see Fig. 5. The threshold probability (x-axis) in this plot encapsulates consideration of the potential surgery side-effects caused by NNSS versus the possibility of having positive surgical margins and disease recurrence, which ultimately depends on the surgeon and patient preference. The net benefit value quantifies the consequences of false positives (FP) and false negatives (FN) in relation to benefit and harm.

In the DCA analysis the risk of side-effects is increased as a consequence of using the model compared to always using the NSS strategy, but the success rate of the surgery is not affected i.e. NNSS and NSS would both have similar chances of successful treatment in a patient that does not have ECE. The CSR model was considered the best model because it achieved the best (or equal) net benefit values for threshold probabilities less than 0.3 on the DCA plot. The assumptions behind the DCA methodology [21] imply that probability thresholds less than 0.3 are equivalent to the assertion that the cost of not using NNSS when ECE is present (i.e. risking failure to achieve curative surgery) is at least 2 1/3 times the cost of causing side-effects by the use of NNSS (2 1/3 = (1–0.3)/0.3). In real-world cases it is likely that this cost ratio would be judged to be larger than 2 1/3 (i.e. the appropriate probability threshold would be < 0.3), and Fig. 5 shows that the CSR model has superior performance over this range.

The mECE variable represents the assessment by radiologist of macroscopic visible extra-prostatic disease on the MR images, and by using this (binary) variable as input to a logistic regression, a model can be built to directly compare the ROC and DCA performance for mECE and the other models. The multivariate models that include semantic and/or radiomics features outperformed the univariate mECE model in terms of AUC (Table 2) and net benefit (for thresholds below 0.3, Fig. 5). In the case of the CS model, this suggests that guiding the radiological assessment by breaking the examination down into more specific factors (i.e. the semantic features) leverages the radiologist’s knowledge more effectively than cognitively summarizing these factors into an overall judgement on the presence of pECE.

Our study has some limitations, the sample size is small and the external validation was performed with external MRI examinations from other institutions, however, interpretated by the same radiologist and operated by the same surgeon. The predictive model is of clinical value to our institution and serves as pilot project, further work will include applying the predictive model to other institutions as the following step approach.

## Conclusion

The combined clinical + semantic + radiomics model can be used to predict pECE + in patients with PCa and results in a positive net benefit when choosing between prostatectomy with NNS or NNSS.

### Electronic supplementary material

Below is the link to the electronic supplementary material.

**Supplementary Material 1: Table S1.** Standardised institutional MR image sequence parameters for Prostate Protocol at 3T. **Table S2.** The inter-reader agreement for MRI semantic features. **Table S3.** The inter-observer variability for radiomics features

## Data Availability

The datasets and models used and/or analysed during the current study are available from the corresponding author on reasonable request.

## Abbreviations

AUC: Area under the curve
DCA: Decision Curve analysis
ECE: Extracapsular extension
NNSS: Non-nerve-sparing surgery
NSS: Nerve-sparing surgery
pECE+: Extracapsular extension in the prostatectomy specimen
