# Supplementary material for: Clinical application of machine learning models in patients with prostate cancer before prostatectomy
Source: Cancer Imaging. 2024 Feb 8;24:24. doi: 10.1186/s40644-024-00666-y (PMC10854130; doi:10.1186/s40644-024-00666-y)
Supplement: Supplementary file 1 — Supplementary Material 1: Table S1. Standardised institutional MR image sequence parameters for Prostate Protocol at 3T. Table S2. The inter-reader agreement for MRI semantic features. Table S3. The inter-observer variability for radiomics features [file 40644_2024_666_MOESM1_ESM.docx]

Supplements Materials

Table S1. Standardised institutional MR image sequence parameters for Prostate Protocol at 3T.

Patients in the discovery dataset was scanned on two 3T scanners (Magnetom Verio and VIDA Siemens Healthcare, Erlangen, Germany) with a pelvic phased-array coil following the standardised institutional protocol according to the Prostate Imaging and Reporting and Data System Version 2 (PIRADS) guidelines (TableS1). The patients in the test dataset were examined using either 1.5T or 3T scanners with a standardized Multiparametric magnetic resonance imaging (mp-MRI) protocol that included T2-weighted Turbo or Fast Spin Echo sequences in all three orthogonal planes, a Diffusion-Weighted Imaging sequence with at least 2 b-values, while the highest b-value was equal or higher than 1000 s/mm2 and lowest b-values 0-50 s/mm2.


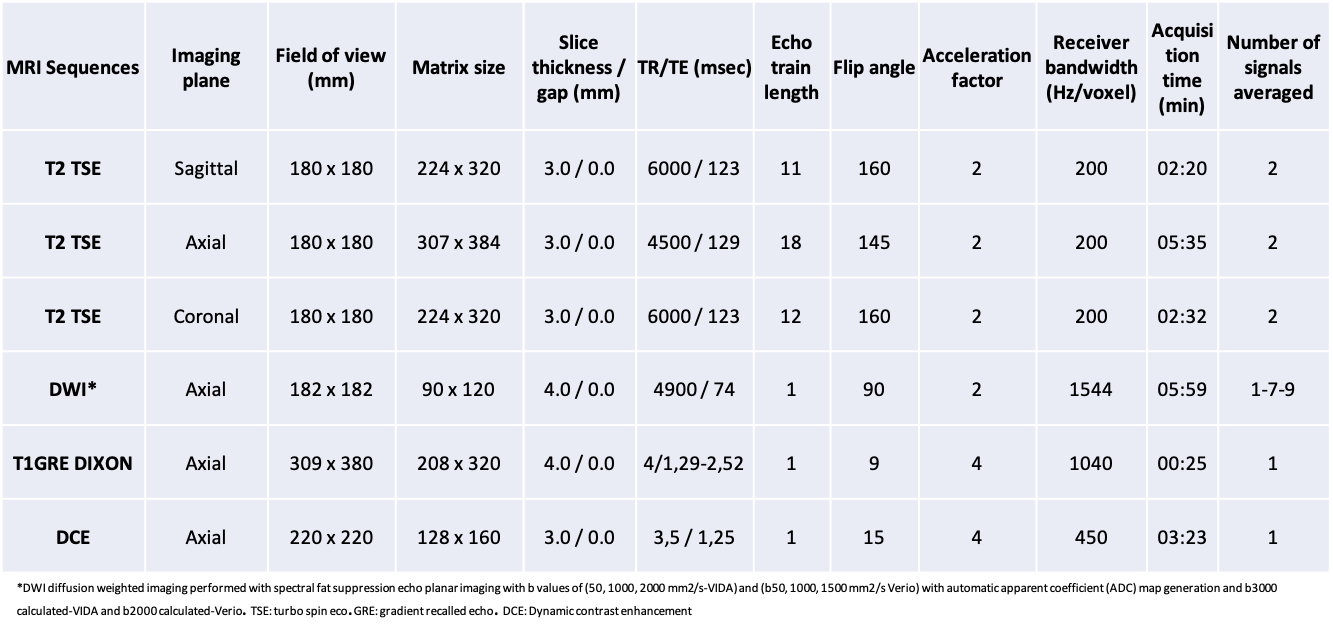
Table S1. Standardised institutional MR image sequence parameters for Prostate

**Table S2. The inter-reader agreement for MRI semantic features.** Inter-observer variability was assessed using the intraclass correlation coefficient (ICC) for continuous semantic features: major length of index lesion and tumour capsular contact length (TCCL), and Cohen's kappa for the others binary semantic features

| **MRI semantic features** | **Reproducibility statistic** |
| --- | --- |
| MajorLengthIndex | 0,683 |
| Tumor Capsular Contact Length | 0,693 |
| SmoothCapsularBulging | 0,152 |
| CapsularDisruption | 0,351 |
| UnsharpMargin | 0,224 |
| IrregularContour | 0,416 |
| Black striation Periprostatic Fat | 0,539 |
| Measurable ECE | 0,786 |
| Retoprostatic angleObliteration | 0,478 |

**Table S3. The inter-observer variability for radiomics features.** It was assessed using the intraclass correlation coefficient (ICC). Radiomic features with ICC>0.75 were used for model building.

| **Feature** | **ICC** |
| --- | --- |
| shape_Elongation | 0,515 |
| shape_Flatness | 0,603 |
| shape_LeastAxisLength | 0,871 |
| shape_MajorAxisLength | 0,846 |
| shape_Maximum2DDiameterColumn | 0,849 |
| shape_Maximum2DDiameterRow | 0,916 |
| shape_Maximum2DDiameterSlice | 0,801 |
| shape_Maximum3DDiameter | 0,885 |
| shape_MeshVolume | 0,904 |
| shape_MinorAxisLength | 0,896 |
| shape_Sphericity | 0,602 |
| shape_SurfaceArea | 0,906 |
| shape_SurfaceVolumeRatio | 0,681 |
| shape_VoxelVolume | 0,904 |
| firstorder_10Percentile | 0,940 |
| firstorder_90Percentile | 0,935 |
| firstorder_Energy | 0,915 |
| firstorder_Entropy | 0,671 |
| firstorder_InterquartileRange | 0,847 |
| firstorder_Kurtosis | 0,343 |
| firstorder_Maximum | 0,709 |
| firstorder_MeanAbsoluteDeviation | 0,776 |
| firstorder_Mean | 0,953 |
| firstorder_Median | 0,959 |
| firstorder_Minimum | 0,773 |
| firstorder_Range | 0,679 |
| firstorder_RobustMeanAbsoluteDeviation | 0,864 |
| firstorder_RootMeanSquared | 0,921 |
| firstorder_Skewness | 0,283 |
| firstorder_TotalEnergy | 0,901 |
| firstorder_Uniformity | 0,709 |
| firstorder_Variance | 0,663 |
| glcm_Autocorrelation | 0,571 |
| glcm_ClusterProminence | 0,489 |
| glcm_ClusterShade | 0,435 |
| glcm_ClusterTendency | 0,715 |
| glcm_Contrast | 0,659 |
| glcm_Correlation | 0,726 |
| glcm_DifferenceAverage | 0,711 |
| glcm_DifferenceEntropy | 0,752 |
| glcm_DifferenceVariance | 0,686 |
| glcm_Id | 0,732 |
| glcm_Idm | 0,724 |
| glcm_Idmn | 0,669 |
| glcm_Idn | 0,719 |
| glcm_Imc1 | 0,767 |
| glcm_Imc2 | 0,845 |
| glcm_InverseVariance | 0,696 |
| glcm_JointAverage | 0,530 |
| glcm_JointEnergy | 0,441 |
| glcm_JointEntropy | 0,534 |
| glcm_MCC | 0,443 |
| glcm_MaximumProbability | 0,480 |
| glcm_SumAverage | 0,530 |
| glcm_SumEntropy | 0,495 |
| glcm_SumSquares | 0,710 |
| glrlm_GrayLevelNonUniformity | 0,888 |
| glrlm_GrayLevelNonUniformityNormalized | 0,718 |
| glrlm_GrayLevelVariance | 0,744 |
| glrlm_HighGrayLevelRunEmphasis | 0,576 |
| glrlm_LongRunEmphasis | 0,743 |
| glrlm_LongRunHighGrayLevelEmphasis | 0,568 |
| glrlm_LongRunLowGrayLevelEmphasis | 0,397 |
| glrlm_LowGrayLevelRunEmphasis | 0,443 |
| glrlm_RunEntropy | 0,570 |
| glrlm_RunLengthNonUniformity | 0,903 |
| glrlm_RunLengthNonUniformityNormalized | 0,739 |
| glrlm_RunPercentage | 0,743 |
| glrlm_RunVariance | 0,738 |
| glrlm_ShortRunEmphasis | 0,741 |
| glrlm_ShortRunHighGrayLevelEmphasis | 0,581 |
| glrlm_ShortRunLowGrayLevelEmphasis | 0,450 |
| glszm_GrayLevelNonUniformity | 0,888 |
| glszm_GrayLevelNonUniformityNormalized | 0,724 |
| glszm_GrayLevelVariance | 0,740 |
| glszm_HighGrayLevelZoneEmphasis | 0,564 |
| glszm_LargeAreaEmphasis | 0,724 |
| glszm_LargeAreaHighGrayLevelEmphasis | 0,644 |
| glszm_LargeAreaLowGrayLevelEmphasis | 0,285 |
| glszm_LowGrayLevelZoneEmphasis | 0,427 |
| glszm_SizeZoneNonUniformity | 0,888 |
| glszm_SizeZoneNonUniformityNormalized | 0,781 |
| glszm_SmallAreaEmphasis | 0,789 |
| glszm_SmallAreaHighGrayLevelEmphasis | 0,599 |
| glszm_SmallAreaLowGrayLevelEmphasis | 0,396 |
| glszm_ZoneEntropy | 0,650 |
| glszm_ZonePercentage | 0,763 |
| glszm_ZoneVariance | 0,675 |
| gldm_DependenceEntropy | 0,669 |
| gldm_DependenceNonUniformity | 0,899 |
| gldm_DependenceNonUniformityNormalized | 0,730 |
| gldm_DependenceVariance | 0,656 |
| gldm_GrayLevelNonUniformity | 0,888 |
| gldm_GrayLevelVariance | 0,743 |
| gldm_HighGrayLevelEmphasis | 0,579 |
| gldm_LargeDependenceEmphasis | 0,722 |
| gldm_LargeDependenceHighGrayLevelEmphasis | 0,634 |
| gldm_LargeDependenceLowGrayLevelEmphasis | 0,197 |
| gldm_LowGrayLevelEmphasis | 0,443 |
| gldm_SmallDependenceEmphasis | 0,768 |
| gldm_SmallDependenceHighGrayLevelEmphasis | 0,641 |
| gldm_SmallDependenceLowGrayLevelEmphasis | 0,447 |
| ngtdm_Busyness | 0,938 |
| ngtdm_Coarseness | 0,838 |
| ngtdm_Complexity | 0,562 |
| ngtdm_Contrast | 0,636 |
| ngtdm_Strength | 0,774 |
